# Switching Stick–Guided Spinal Needle Repair Technique for Small Size Rotator Cuff Tear

**DOI:** 10.1016/j.eats.2024.103309

**Published:** 2024-11-18

**Authors:** Thitiphol Wanitchanont, Peerapat Lertwiram, Chaiyanun Vijittrakarnrung

**Affiliations:** a Chakri Naruebodindra Medical Institute, Faculty of Medicine Ramathibodi Hospital, Mahidol University, Samut Prakan, Thailand; b Department of Orthopedics, Faculty of Medicine Ramathibodi Hospital, Mahidol University, Bangkok, Thailand

## Abstract

Symptomatic small rotator cuff tears are a common issue for orthopedic surgeons. While arthroscopic repair is the standard treatment after conservative methods fail, no consensus exists on the ideal technique. This study presents a rotator cuff tissue-preserving repair method using a switching stick and spinal needle, which is reproducible, easy to learn, and ensures equal tendon tissue tension post-repair.

Rotator cuff tendon tears are a leading cause of shoulder pain in adults, particularly in the aging population.^1^ Tears are commonly classified by size and tear extension.^2^, ^3^, ^4^ Although previous studies have shown comparable short- to medium-term outcomes between conservative and surgical management for small tears, surgery remains the gold standard for cases unresponsive to conservative treatment. Notably, a randomized controlled trial by Moosmayer et al. demonstrated significantly better outcomes for surgical repair of small cuff tears compared with nonsurgical treatment at a 10-year follow-up.^5^ Furthermore, untreated tears carry the risk of progression and clinical deterioration.^6^ In contemporary practice, arthroscopic rotator cuff repair has become the standard treatment for such injuries. The success of these repairs is influenced by several factors, including patient age, tendon quality, tear size, equipment, and surgical expertise.^7^ Among these, the chosen repair technique stands out as a critical determinant of success.

This study introduces an arthroscopic technique for repairing small rotator cuff tears, encompassing high-grade partial- and full-thickness tears, using a switching stick and a spinal needle. In all these tears, one of the main challenges is visualizing the small bony footprint, which is frequently hidden by the remaining rotator cuff tissue. We believe that our proposed technique can enhance repair accuracy, preserve more rotator cuff tissue, and achieve near-anatomic approximation of the tears while avoiding tension length mismatch compared with conventional repairing technique.

## Surgical Technique

### Intra-Articular Management

**Fig 1:**
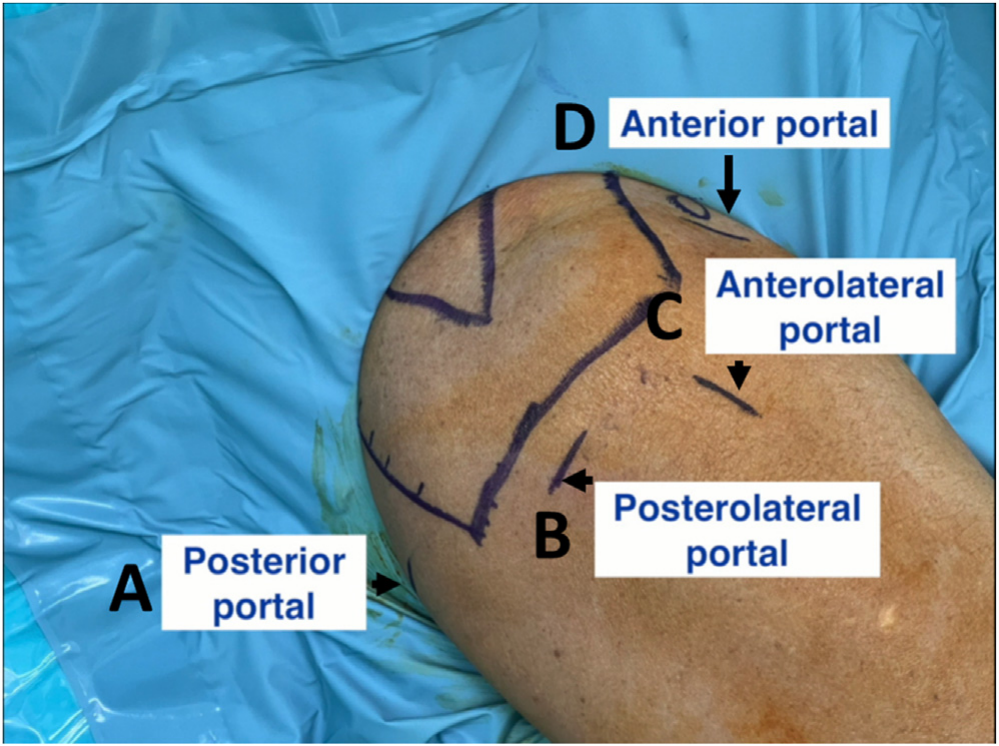
Patient is placed in the beach-chair position with a sterile shoulder drape. The anatomic landmarks and 4 portals are drawn on the right shoulder. (A) A standard posterior viewing portal. (B) A posterolateral portal. (C) An anterolateral portal. (D) An anterior working portal.

The anatomic landmark is drawn (Fig 1). Video 1 demonstrates the surgical steps. Pearl and pitfalls are summarized in Table. 1. A routine arthroscopic examination is done through the posterior viewing portal, and the anterior working portal is created using the outside-in technique. Debridement and, if needed, tenodesis or tenotomy of the biceps are performed.

**Table 1:** Pearl and Pitfalls

**Pearls**
• Preoperative magnetic resonance imaging evaluation of tear location is essential for accurate intraoperative identification.
• Visualization of the intra-articular aspect of a supraspinatus tear can be improved by positioning the arm in abduction and external rotation through a posterior viewing portal.
• Adequate subacromial bursectomy is necessary for clear visualization and understanding of the tear configuration.
• Perform an acromioplasty, if necessary, to create sufficient space for needle trajectory repair.
• Use an arthroscopic shaver to debride degenerate tendon tissue because radiofrequency ablation may damage healthy tissues.
**Pitfalls**
• Incomplete removal of soft tissue and bursa can obstruct visualization and prolong surgical time.
• This technique is suitable only for small tears; for cases with medium to large ruptures, a retracted tear may not be adequately captured by the spinal needle.
• Surgeons may experience a learning curve if they are accustomed to viewing the rotator cuff from the subacromial space.

The anterior articular portion of the supraspinatus tendon is identified and examined. Visualization improves with the shoulder in abduction and external rotation. The affected area is located outside-in using an 18-gauge spinal needle, guided by preoperative magnetic resonance imaging geographically relative to the biceps tendon, and a polydioxanone suture (PDS) is passed through the needle and retrieved through the anterior portal.

### Subacromial Management

**Fig 2:**
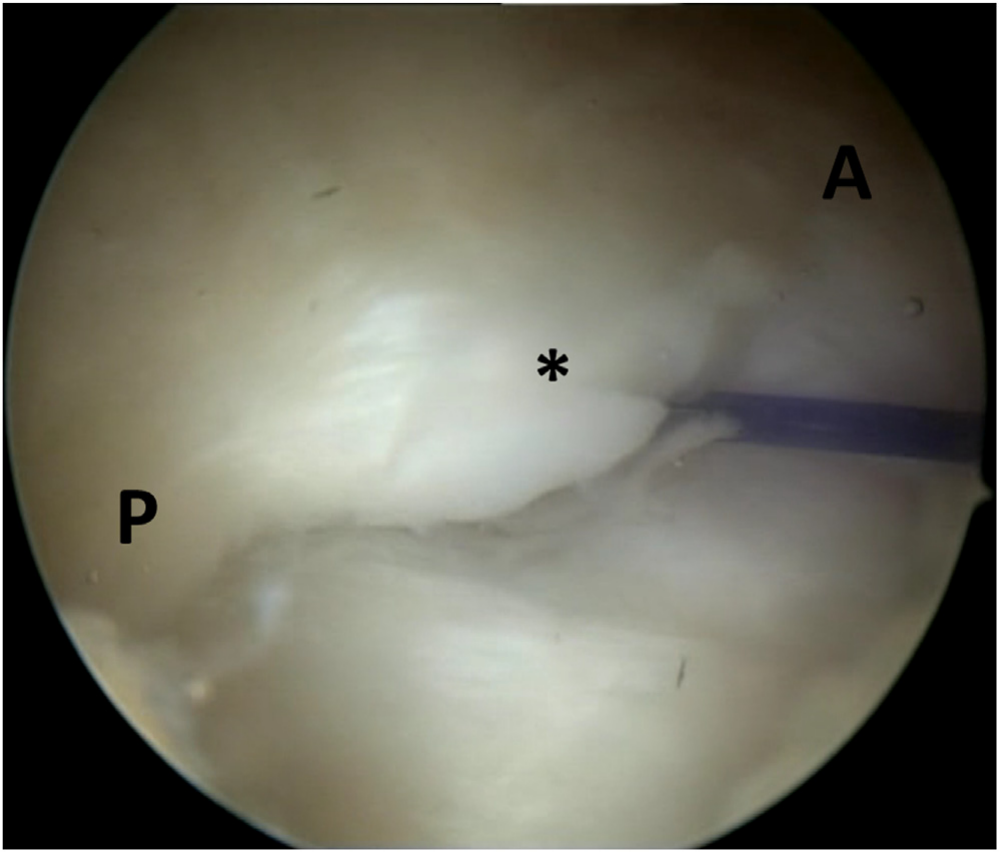
During the arthroscopic procedure on the right shoulder, with the patient positioned in the beach-chair position, a subacromial view through the posterolateral portal is obtained. The small bony footprint of supraspinatus is obscured by the remaining healthy cuff tissue (asterisk). A, anterior; P, posterior.

The subacromial space is accessed to confirm the tear site and evaluate the bursal side of the rotator cuff. An anterolateral portal is created near the PDS entry point at the acromial edge. Subacromial debridement, bursectomy, and acromioplasty are performed as needed. Once the tear site is identified, the edges are carefully debrided to expose healthy tendon tissue. Preserving healthy cuff tissue can make visualizing the cuff footprint challenging. In many cases of small cuff tears, the small bony footprint is obscured by the remaining healthy tissue, complicating accurate anchor placement and the use of other suture passers from subacromial space (Fig 2).

### Switching Stick–Guided Spinal Needle Repair Technique

**Fig 3:**
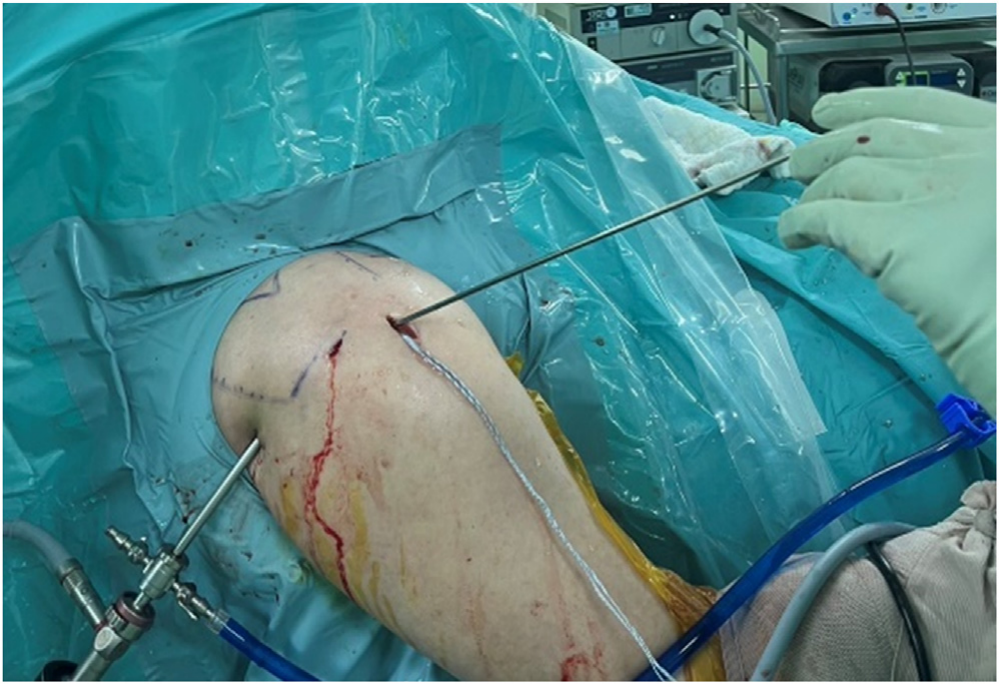
During the arthroscopic procedure on the right shoulder, positioned in the beach chair orientation. The switching stick is inserted into tear site via anterolateral portal to confirm the location and direction of the tear.

The intra-articular space is accessed again, and the switching stick is guided to the tear site via the anterolateral portal, following the spinal needle’s path (Fig 3). An all-suture anchor (2.8 Y-Knot RC with Tape, Conmed-Linvatec, Largo, FL) is placed near the articular cartilage edge, viewed from the posterior portal. If inserting the anchor through the tear is difficult, a slotted or half-pipe cannula is used.

**Fig 4:**
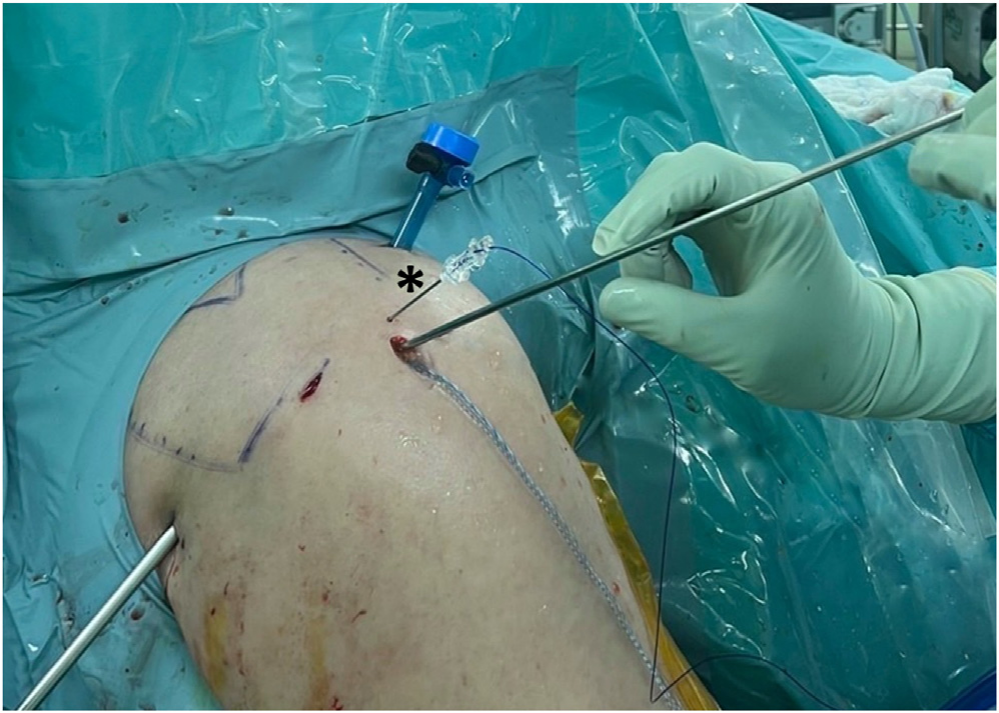
Arthroscopic procedure on the right shoulder, with the patient positioned in the beach-chair position. An 18-gauge spinal needle (18G) with polydioxanone suture is pierced through the skin near the anterolateral portal directed to the tip of switching stick using triangular technique (asterisk).

**Fig 5:**
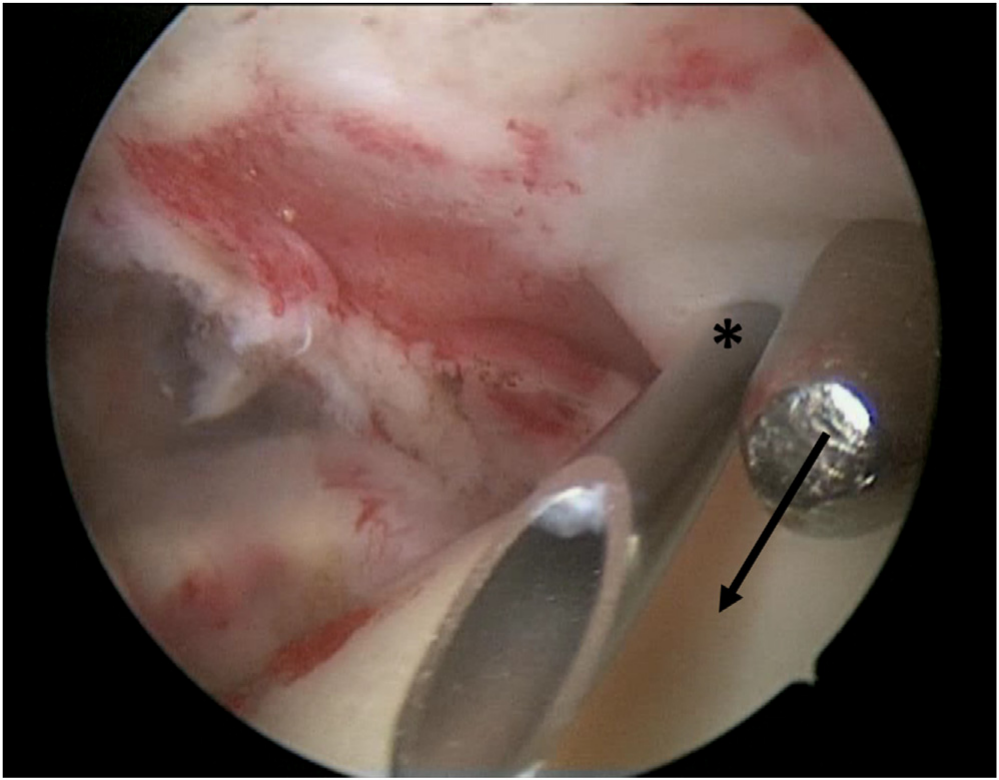
During the arthroscopic procedure on the right shoulder, with patient positioned in the beach-chair position, an intra-articular view through the posterior portal is obtained. The locations of needle placement are confirmed (asterisk), with preferred suture locations 3-5 mm medial to the edge of the torn tendon, guided directionally by the switching stick (arrow).

The switching stick is reinserted into the tear site, maintaining the same direction. An 18-gauge spinal needle with PDS is inserted near the anterolateral portal, 1 to 1.5 cm apart, and directed to the switching stick using a triangular technique (Fig 4). Suture placement is confirmed through the posterior portal, ideally 3 to 5 mm medial to the torn tendon edge (Fig 5). The switching stick is then removed, and the PDS is passed through the spinal needle and retrieved out the anterior portal. The spinal needle is then removed.

**Fig 6:**
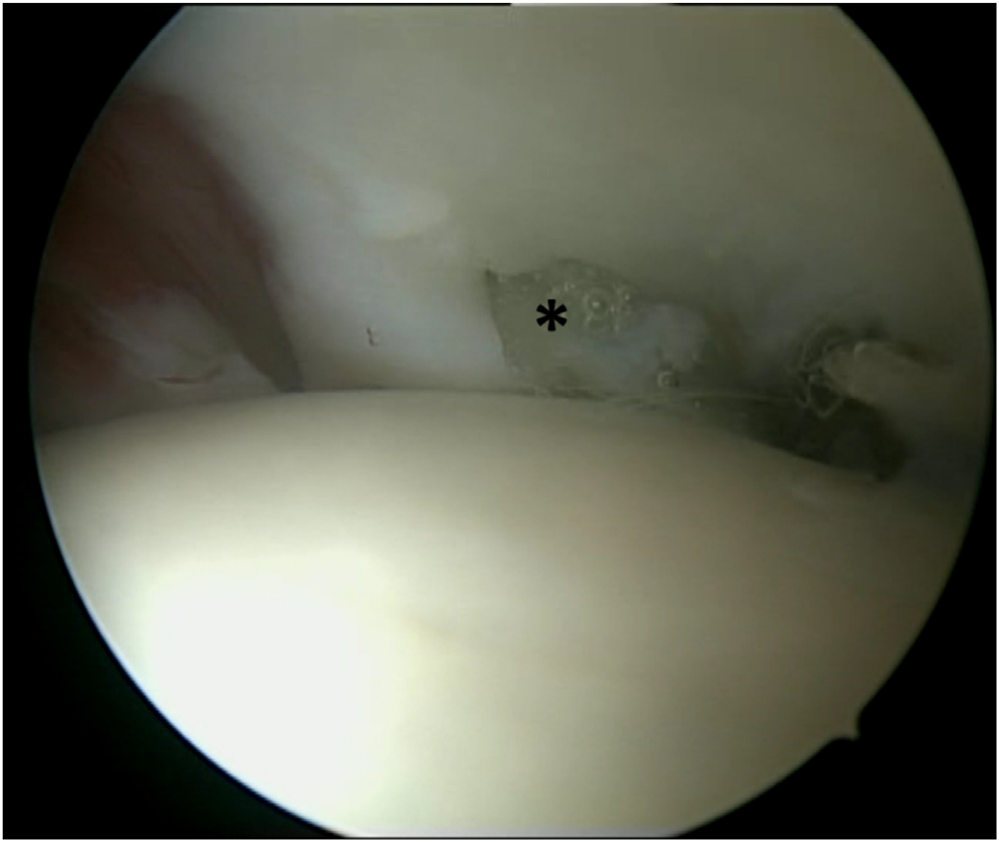
During the arthroscopic procedure on the right shoulder, positioned in the beach chair orientation, an intra-articular view through the posterior portal was obtained. The suture limb is pulled out by polydioxanone suture at the skin near anterolateral portal (asterisk).

**Fig 7:**
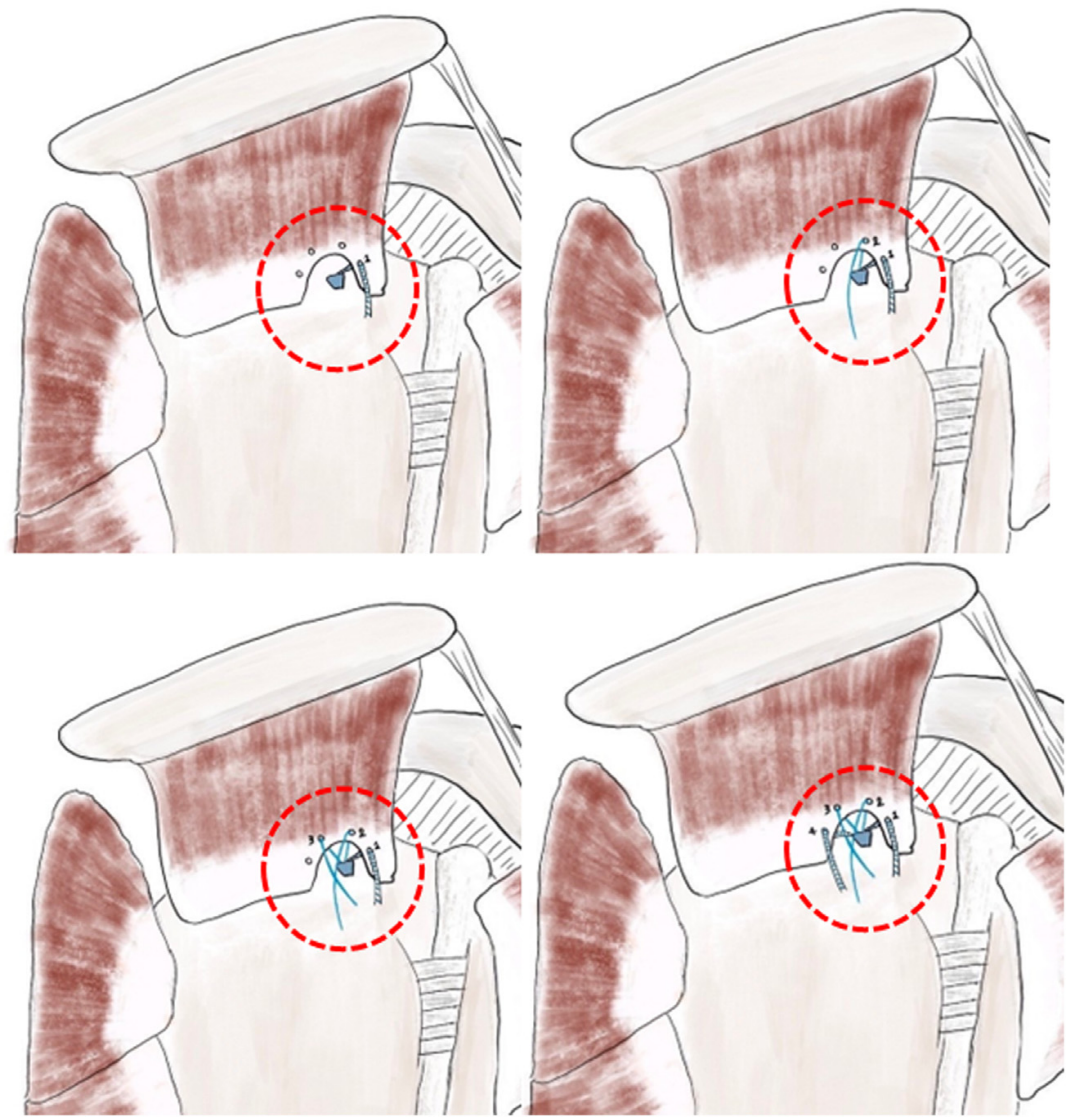
This needle is repeated for all-suture limbs using a parachute configuration pattern over the rotator cuff tendon (circle).

The suture limb is passed from the anchor into the joint with a suture retriever and is retrieved through the anterior portal. The suture limb is then placed into the PDS loop, which is pulled out near the anterolateral portal (Fig 6). This process is repeated for all suture limbs using a parachute configuration (Fig 7). The subacromial space is checked to ensure proper suture placement, using a posterolateral portal if necessary. The 2 middle suture limbs are tied as preferred, and all limbs are secured into a knotless anchor at the lateral row (3.5 Poplok, Conmed-Linvatec).

For high-grade articular-side tears needing repair, conversion to full thickness is considered based on the tear location and tissue quality. For tears at the posterior footprint, a longitudinal split of the supraspinatus tendon is made with a surgical blade or hook electrocautery, followed by anchor placement and repair. For tears at the anterior footprint, the outer layer of the supraspinatus tendon is preserved, with anchor placement done from the anterior portal. In this case, an 18-gauge spinal needle, rather than the switching stick, confirms the tear’s location and direction.

### Postoperative Rehabilitation

After surgery, the shoulder is immobilized in a sling for 6 weeks. Pendulum exercises start immediately, with active motion at 6 weeks and strengthening exercises later. The goal is pain-free daily movement, full motion by 3 months, and strengthening by 6 months.

## Discussion

The optimal treatment for small rotator cuff tears remains a subject of debate. Conservative approaches are generally recommended as initial steps. Surgical repair is typically considered when a tear exceeds 50% of the thickness on either the bursal or articular side.^8^

Currently, there is no consensus on the ideal repair technique for arthroscopic repair of small-sized rotator cuff tears, especially high-grade partial tears.^9^ Two surgical techniques are commonly used including the in situ transtendinous repair involves repairing the torn cuff tissue to the footprint while preserving the remaining tissue, and the tear completion repair involves detaching the remnant cuff tissue and reattaching it to the original footprint. The latter technique can result in potential tension mismatches due to inaccurate suture placement. Although biomechanical studies on animal models have demonstrated that tear completion repair offers superior biomechanical and histological outcomes,^10^, ^11^, ^12^ a systematic review by Prasetia et al. revealed no significant difference in clinical outcomes between tear completion and in situ repair techniques. However, the review did note that in situ repair is associated with a higher rate of retear.^9^ Furthermore, research by Jordan et al. indicated that transtendinous repairs result in greater pain and poorer function during the first 3 postoperative months.^13^

To maximize the benefits and minimize the drawbacks of tear completion repair, our technique offers several advantages over conventional rotator cuff repair methods (Table 2). Traditional approaches involve suturing the tendon using suture-passing devices in either a retrograde or antegrade fashion from the subacromial aspect, with diameters ranging from 1.5 to 4 mm.^14^ In cases of small rotator cuff tears, the inner layer hole is smaller than the outer layer, requiring additional debridement of the inner layer and superior capsule to accommodate these devices. Our method, however, uses an 18-gauge spinal needle with a diameter of only 1 mm, thereby minimizing tendon tissue trauma.

**Table 2:** Advantages and Risks

**Advantages**
• Less tendon tissue trauma because the diameter of the spinal needle is 1 mm
• Greater preservation of the superior capsule and inner layer of supraspinatus tendon tissue
• Ensure accurate placement of intra-articular sutures to achieve proper reduction of the superior capsule and the inner layer of the supraspinatus tendon
• Less dog-ear deformity and equal tension of tendon fiber
• Accurate anchor placement from direct visualization intra-articularly
**Risks**
• Missed outer layer of supraspinatus tendon in larger or retracted cuff tissue tendon tears
• Tissue trauma from repeated needle insertion with improper needle placement
• No biomechanical investigations are currently available for the proposed repair technique

Additionally, our technique preserves the inner layer of tendon tissue and the superior capsule by using a switching stick to locate the tear site intra-articularly and guide the repair direction. The switching stick, with a diameter of only 5 mm, is significantly smaller compared with other suture-passing devices.^15^ Furthermore, antegrade suture-passing devices often do not allow precise intra-articular placement, frequently resulting in sutures that are too far or too near the torn intra-articular tendon edge. Auto-retrieving suture-passing devices deploy needles at a fixed distance of 5-7 mm, which can cause tension mismatches and deformities such as dog-ear folds in the repaired tendon.^16^ Our technique ensures precise suture placement 3 to 5 mm from the tendon edge, securing adequate tissue from the superior capsule and the deep layer of the supraspinatus tendon because the viewing portal is intra-articular.

Lastly, conventional anchor placement is typically viewed from the subacromial space, where the anchor itself can obscure its placement location, sometimes resulting in slippage and misalignment with the edge of the articular cartilage. This method allows accurate confirmation of anchor placement by viewing from the intra-articular space, ensuring proper alignment and attachment.

This technique has some limitations. The spinal needle’s angle and placement are constrained by the acromion’s lateral projection. In larger or retracted tears, the needle may not capture the tendon’s outer layer, requiring a suture retriever or hanging suture to pull the tendon to its footprint. Additionally, blindly piercing the supraspinatus tendon with the spinal needle can risk tissue damage if the needle is improperly placed. The switching stick–guided spinal needle technique offers significant benefits in repairing small rotator cuff tears, notably preserving cuff tissue and preventing tension mismatches postrepair. This cost-effective method is suitable for all small rotator cuff tears, including high-grade partial thickness and full-thickness tears.

## Disclosures

The authors (T.W., P.L., C.V.) declare that they have no known competing financial interests or personal relationships that could have appeared to influence the work reported in this paper.

## Supplementary Data

Video 1 This video features a case presentation of a high-grade bursal-side supraspinatus tear that failed conservative treatment. The surgical technique of switching stick–guided spinal needle repair for a small rotator cuff tear, performed in the beach-chair position on the right shoulder, is shown. An anatomic landmark is drawn, and the arthroscopic examination is performed. Debridement, and if necessary, tenodesis or tenotomy of the biceps, are done. The supraspinatus tendon is identified and visualized in abduction and external rotation, and the affected area is located with a spinal needle for suture placement. In the subacromial space, tear site identification and debridement are performed through an anterolateral portal. The switching stick–guided spinal needle technique is used for all suture passage. The suture limbs are secured in a parachute configuration, with final knots tied and fixed into a knotless anchor.

## References

[1] Hinsley H., Ganderton C., Arden N.K., Carr A.J. (2022). Prevalence of rotator cuff tendon tears and symptoms in a Chingford general population cohort, and the resultant impact on UK health services: A cross-sectional observational study. BMJ Open.

[2] Cofield R.H. (1985). Rotator cuff disease of the shoulder. J Bone Joint Surg Am.

[3] Davidson J., Burkhart S.S. (2010). The geometric classification of rotator cuff tears: A system linking tear pattern to treatment and prognosis. Arthroscopy.

[4] Lädermann A., Denard P.J., Collin P. (2015). Massive rotator cuff tears: definition and treatment. Int Orthop.

[5] Moosmayer S., Lund G., Seljom U.S. (2019). At a 10-year follow-up, tendon repair is superior to physiotherapy in the treatment of small and medium-sized rotator cuff tears. J Bone Joint Surg Am.

[6] Quinlan N.J., Frandsen J.J., Smith K.M., Lu C.C., Chalmers P.N., Tashjian R.Z. (2022). Conservatively treated symptomatic rotator cuff tendinopathy may progress to a tear. Arthrosc Sports Med Rehabil.

[7] Danilkowicz R., Levin J.M., Crook B., Long J.S., Vap A. (2022). Analysis of risk factors, complications, reoperations, and demographics associated with open and arthroscopic rotator cuff repair: An analysis of a large national database. Arthroscopy.

[8] Kim Y.S., Lee H.J., Bae S.H., Jin H., Song H.S. (2015). Outcome comparison between in situ repair versus tear completion repair for partial thickness rotator cuff tears. Arthroscopy.

[9] Prasetia R., Kholinne E., Suvarly P. (2021). High-grade bursal side rotator-cuff repair: A surgical outcome review. Orthop Res Rev.

[10] Pulatkan A., Anwar W., Ayık O. (2020). Tear completion versus in situ repair for 50% partial-thickness bursal-side rotator cuff tears: A biomechanical and histological study in an animal model. Am J Sports Med.

[11] Gereli A., Kocaoglu B., Ulku T.K. (2018). Completion repair exhibits increased healing characteristics compared with in situ repair of partial thickness bursal rotator cuff tears. Knee Surg Sports Traumatol Arthrosc.

[12] Ranalletta M., Rossi L.A., Atala N.A., Bertona A., Maignon G.D., Bongiovanni S.L. (2017). Arthroscopic in situ repair of partial bursal rotator cuff tears without acromioplasty. Arthroscopy.

[13] Jordan R.W., Bentick K., Saithna A. (2018). Transtendinous repair of partial articular sided supraspinatus tears is associated with higher rates of stiffness and significantly inferior early functional scores than tear completion and repair: A systematic review. Orthop Traumatol Surg Res.

[14] Chokshi B.V., Kubiak E.N., Jazrawi L.M. (2006). The effect of arthroscopic suture passing instruments on rotator cuff damage and repair strength. Bull Hosp Joint Dis.

[15] Kim M.S., Kim D.W., Choi Y.E., Bachman L., Kim S.H. (2015). Performance of antegrade suture passers according to tendon thickness. Int J Shoulder Surg.

[16] Owen M.T., Loy B.N., Guttmann D., Reid J.B. (2020). Prevention, reduction, and stabilization of dog-ear deformities during arthroscopic rotator cuff repair. Arthrosc Tech.

